# Individual alpha frequency appears unrelated to the latency of early visual responses

**DOI:** 10.3389/fnins.2023.1118910

**Published:** 2023-04-11

**Authors:** Audrey Morrow, Wei Dou, Jason Samaha

**Affiliations:** Department of Psychology, University of California, Santa Cruz, Santa Cruz, CA, United States

**Keywords:** alpha frequency, discrete sampling, rhythmic perception, C1 ERP, N150 ERP

## Abstract

A large body of work has linked neural oscillations in the alpha-band (8–13 Hz) to visual perceptual outcomes. In particular, studies have found that alpha phase prior to stimulus onset predicts stimulus detection, and sensory responses and that the frequency of alpha can predict temporal properties of perception. These findings have bolstered the idea that alpha-band oscillations reflect rhythmic sampling of visual information, however the mechanisms of this are unclear. Recently two contrasting hypotheses have been proposed. According to the *rhythmic perception* account, alpha oscillations impose phasic inhibition on perceptual processing and primarily modulate the amplitude or strength of visual responses and thus the likelihood of stimulus detection. On the other hand, the *discrete perception* account proposes that alpha activity discretizes perceptual inputs thereby reorganizing the timing (not only the strength) of perceptual and neural processes. In this paper, we sought neural evidence for the discrete perception account by assessing the correlation between individual alpha frequencies (IAF) and the latency of early visual evoked event-related potential (ERP) components. If alpha cycles were responsible for shifting neural events in time, then we may expect higher alpha frequencies to be associated with earlier afferent visual ERPs. Participants viewed large checkerboard stimuli presented to either the upper or lower visual field that were designed to elicit a large C1 ERP response (thought to index feedforward primary visual cortex activation). We found no reliable correlation between IAF and the C1 latency, or subsequent ERP component latencies, suggesting that the timing of these visual-evoked potentials was not modulated by alpha frequency. Our results thus fail to find evidence for discrete perception at the level of early visual responses but leave open the possibility of rhythmic perception.

## Introduction

Brain dynamics in the alpha-band (8–13 Hz) have been shown to predict various aspects of visual perception, such as the probability of target detection as well as temporal properties of perception. Although there is much evidence to support the involvement of alpha *power* in the suppression of neural activity and perceptual reports [reviewed in Samaha et al. (2020)] as well as growing evidence regarding the relevance of alpha *phase* (VanRullen, 2016), it is less clear how alpha frequency may be involved in shaping visual information processing. Two theories have been proposed for how alpha-band frequency dynamics may relate to variations in visual perception: the rhythmic perception account and the discrete perception account (VanRullen, 2016). The rhythmic perception account proposes that alpha oscillations reflect phasic changes in neuronal excitability which principally modulate the *intensity* of perception and/or sensory responses. On the other hand, the discrete perception account suggests that alpha oscillations are involved in the *timing* and discretization of sensory events. According to these accounts, an individual’s alpha frequency would either be related to the frequency and duration of excitability changes (rhythmic perception) or the discretization rate of perception (discrete perception). As recent reviews (Kasten and Herrmann, 2022; Menétrey et al., 2022) and experiments (Morrow and Samaha, 2022) have pointed out, current evidence does not clearly support one account over the other.

Studies have demonstrated a relationship between an individual’s peak alpha frequency (IAF) and temporal properties of their perception, but have not necessarily disentangled rhythmic from discrete perception. For example, several studies have linked variation in IAF to the temporal resolution of visual (Coffin and Ganz, 1977; Samaha and Postle, 2015; Baumgarten et al., 2018; Gray and Emmanouil, 2020) and multisensory perception (Cecere et al., 2015; Cooke et al., 2019; Migliorati et al., 2020; Noguchi, 2022; but see Buergers and Noppeney, 2022), typically finding that higher alpha frequencies correspond to shorter windows of integration. These experiments, however, do not rule out intensity-based accounts whereby IAF is related to the duration of the period of excitation and inhibition. For instance, according to the rhythmic perception account, one of the stimuli in each trial (or the gap between stimuli) may be more likely to be missed (rather than integrated) due to a longer integration window (Fan, 2018). This account would be consistent with a growing body of literature demonstrating that alpha-band phase modulates perceptual detection (Busch et al., 2009; Mathewson et al., 2009; Dugué et al., 2011; Ai and Ro, 2014; Samaha et al., 2015, 2017; Alexander et al., 2020) and neuronal responses (Haegens et al., 2011; Spaak et al., 2014; Dougherty et al., 2017; Dou et al., 2022).

Here, we sought neural evidence for the hypothesis that IAF modulates the timing of sensory processes, which would be consistent with the discrete perception account. Specifically, we examined whether individual differences in alpha frequency predict the timing of early visual responses with a focus on the striate and extrastriate visual evoked potentials. We examined data from two studies that used high-contrast checkerboard stimuli, which are known to elicit large C1 event-related potential (ERP) responses. We extracted IAF from a prestimulus window in order to assess whether the frequency of prestimulus alpha-band activity modulates the onset and peak latency of early visual-evoked potentials. If alpha frequency is related to the discretization of visual perception, then we might expect higher frequencies to be associated with earlier onset sensory responses (i.e., quicker perceptual updates).

## Method

### EEG Datasets, Stimuli, and Tasks

#### Task-irrelevant viewing

**Figure 1:**
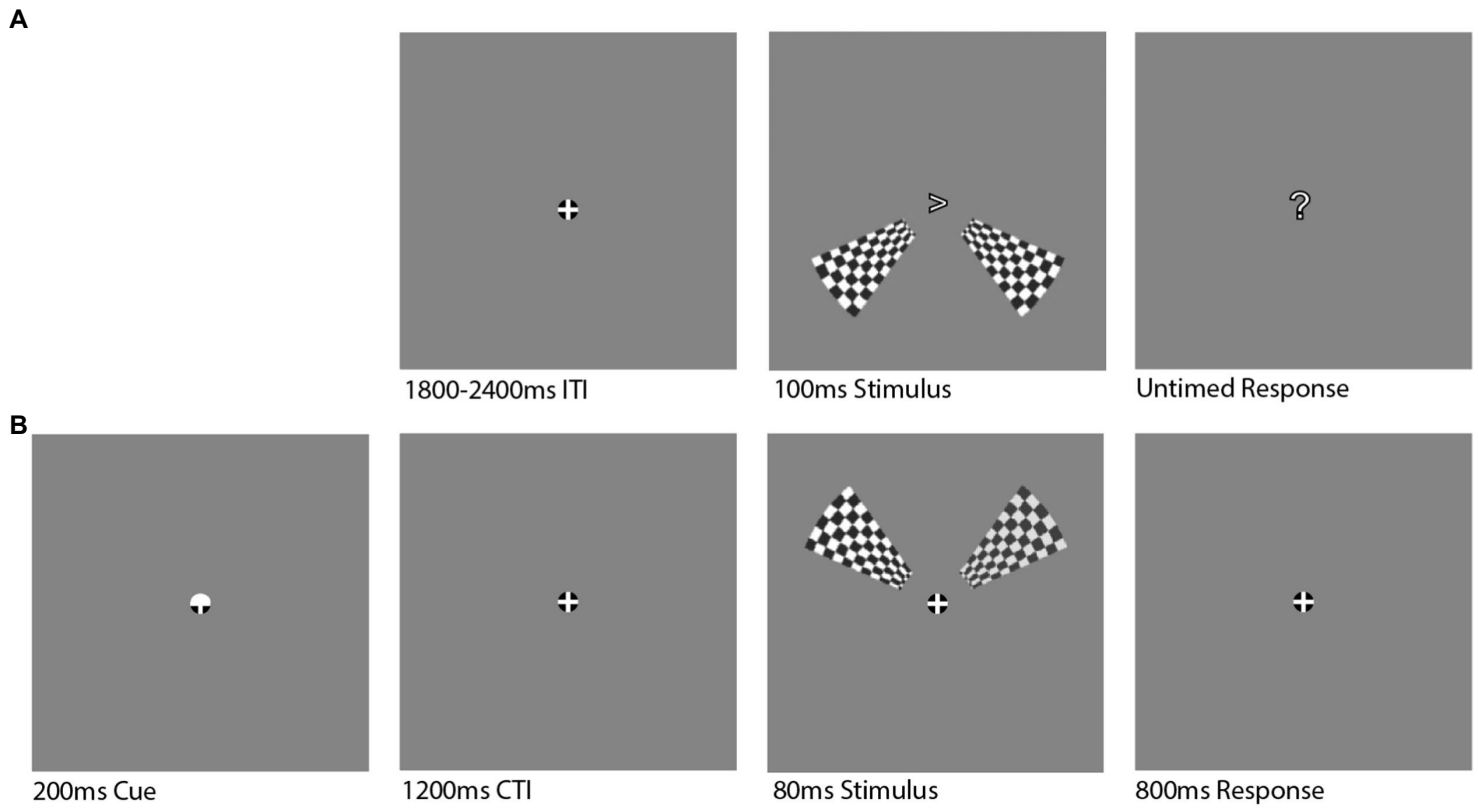
Stimulus Design. Data sets were analyzed from two different experimental designs that both presented large checkerboard wedge stimuli to either the upper or lower visual field to evoke a large C1 component response. **(A)** In the task-irrelevant viewing paradigm, a fixation was presented for a variable inter-trial interval (ITI) and then two full contrast checkerboard wedges were presented for 100 ms to either the upper or lower (pictured) visual field. A question mark then appeared to signal to the participant to push a button with their dominant hand to indicate which direction a fixation arrow had pointed during the stimulus presentation. **(B)** In the spatial attention task, a cue highlighted either the upper or lower half of the fixation for 200 ms and indicated to participants to covertly shift their attention to the upper or lower visual field. After a 1,200 ms cue-target interval (CTI), the same two checkerboard wedges were presented for 80 ms, except the right wedge varied in contrast between 60 and 100% while the left wedge was held at 80% contrast. Participants were then asked to indicate which wedge had a greater level of contrast *via* button press (“<” or “>” for left or right, respectively).

**Figure 2:**
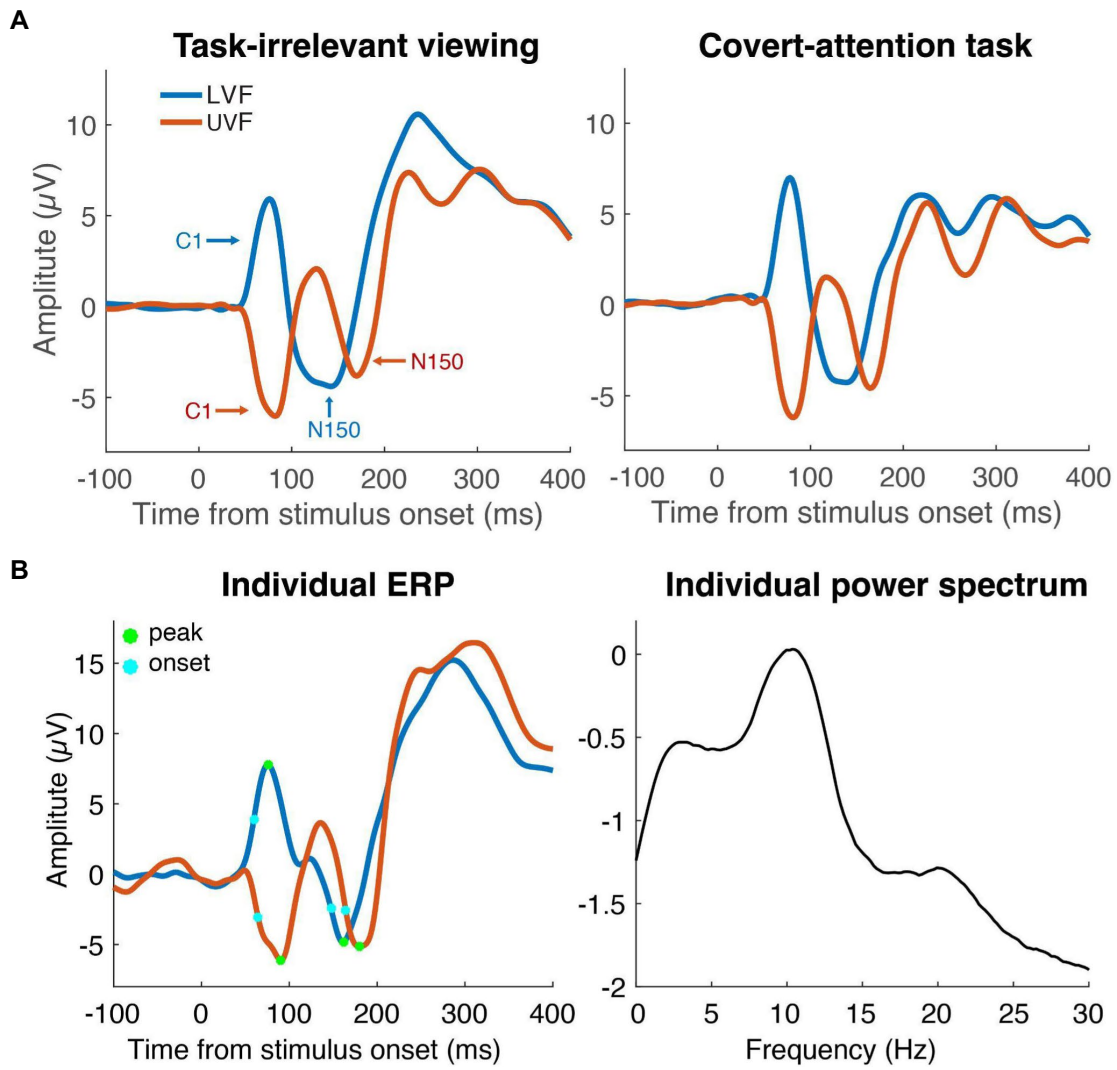
Event-Related Potentials (ERPs). **(A)** Average ERPs of LVF (blue line) and UVF (red line) stimuli in the task-irrelevant viewing dataset (left) and the covert-attention task (right). ERPs were recorded from the individual electrode with the largest C1 component. The C1 peaked around 90 ms and the N150 peaked within the time window of 100 to 200 ms. **(B)** Average ERPs (left) of one individual participant with points showing the onset latency (light blue dots) and peak latency (green dots) of the C1 and N150 components. The same participant’s power spectrum from pre-stimulus data is shown in the right panel. Peak frequency was defined as the frequency with largest amplitude in the range form 7–14 Hz.

Two electroencephalogram (EEG) datasets were analyzed in this study due to the comparable stimuli used across the two experimental designs. The first dataset comes from a study conducted by Iemi et al. (2019) and is available for download at https://osf.io/yn6gb/. The data were collected from 27 participants (M_age_ = 26.33, *SEM* = 0.616; 14 females) with normal or corrected vision, although three participants’ data were excluded from the analysis because they either did not finish the experiment or did not exhibit the C1 component in the LVF. In the original experiment, a pair of task-irrelevant, full-contrast checkerboard wedges were presented for 100 ms in either the upper (UVF) or lower visual field (LVF; Figure 1). These wedges were designed with spatial frequency, location, and size characteristics that should activate the primary visual cortex in both hemispheres and produce a constructive summation of electrical fields, resulting in robust C1 responses (Figure 2). Participants were presented with an arrow at fixation indicating leftward or rightward direction while the task-irrelevant checkerboard stimuli were presented in UVF or LVF with equal probability. Participants were tasked with reporting the direction of the central arrow using the “<” or “>” key for left or right, respectively, while ignoring the checkerboards. Experimental blocks were 90 trials each with 60 stimulus-present trials and 30 stimulus-absent trials randomly distributed. Participants completed 9 blocks, totaling 810 trials. This dataset was collected from 64 channels arranged according to the International 10–10 system using a BioSemi ActiveTwo system with a 1,024 Hz sampling rate. All channels were referenced online to the CMS-DRL ground electrodes. More detail of experimental procedures can be found in the original study (Iemi et al., 2019).

#### Covert-attention task

The second dataset comes from a cued spatial attention experiment that was conducted in our lab which has not yet been published. This study was approved by the Institutional Review Board of University of California Santa Cruz (UCSC). 21 participants from the UCSC community completed the experiment (M_age_ = 22; *SEM* = 0.941; 15 female, 4 male, 1 non-binary, and 1 undisclosed). Three participants were excluded from the analysis for either poor task accuracy (1 participant) or the lack of a clear C1 ERP response (2 participants). All participants had normal or corrected-to-normal vision and were compensated with course credit and a $20 gift card. Data and task scripts can be found at https://osf.io/5egkp/.

This cued attention experiment used an adapted version of the checkerboard stimuli for a covert-attention cued contrast discrimination task. This task also presented the two checkerboard wedges to either the upper or lower visual field, but participants were instructed to attend to the cued visual field and report which wedge had a greater contrast. The right wedge was fixed at 80% contrast while the left wedge varied between 60 and 100% contrast. On each trial, a fixation consisting of a white cross centered within a black circle of 0.5 degrees of visual angle (DVA) on a 50% gray background was present on the screen for the duration of the experiment. The top or bottom of the fixation circle turned white for 200 ms to indicate for participants to shift their attention to the cued visual field. After a cue-target interval of 1,200 ms, the two bilateral checkerboard wedges were presented to either the upper or lower visual field. The stimulus code was copied from Iemi et al. (2019) and consisted of wedge segments taken from a radial checkerboard pattern with 15 circles and 68 radial lines, with the first, inner circle beginning 3 DVA from central fixation and the final, outer circle ending 10 DVA from central fixation. The wedges were presented for 80 ms and participants had 800 ms to respond with “<” or “>” button press according to whether they perceived the left or right wedge to have a great amount of contrast, respectively. The right edge was always presented at 80% contrast and the left wedge varied in contrast such that it was randomly presented at either 60, 100% or one of eight linearly-spaced contrast levels between 74 and 86% contrast. Participants completed 10 blocks of 100 trials each, within which the cue was valid 80% of the time. This second dataset was recorded from 64 electrodes corresponding to the International 10–10 system using an actiCHamp EEG system with a 1,000 Hz sampling rate. All channels were referenced online to channel “Cz.” The stimulus presentation was controlled by Psychtoolbox 3 (Pelli, 1997; Kleiner et al., 2007) running in the MATLAB environment on an Ubuntu operating system.

### EEG Preprocessing

Raw data from both datasets were preprocessed in the same way using custom Matlab scripts in conjunction with EEGLAB toolbox functions (Delorme and Makeig, 2004). Datasets were high-pass filtered at 0.1 Hz and low-pass filtered at 40 Hz using a zero-phase Hamming-windowed sinc FIR filter, downsampled to 500 Hz, and epoched to include trial data from 2 s before through 2 s after stimulus onset. The data were manually inspected to remove trials and channels with artifacts such as muscle movement or eye blinks that overlapped with stimulus presentation. Noisy channels were interpolated and an independent components analysis using the INFOMAX algorithm (EEGLAB function *binica.m*) was used to remove ocular artifacts. For the task-irrelevant viewing dataset, an average of 0.58 electrodes were interpolated using spherical spline interpolation and an average of 30.46 trials were rejected for each participant. For the covert attention dataset an average of 3.4 electrodes were interpolated using spherical spline interpolation and an average of 164.6 trials were rejected for each participant. Data were then re-referenced to the average of all channels and baseline corrected using a 200 ms prestimulus baseline window.

### Analysis

The goal of our study was to extract the IAF for each subject along with the timing of their early sensory responses (with a focus on the C1 component). To this end, we first identified the electrode for each subject that had the largest C1 amplitudes for upper and lower visual fields. For 73% of subjects, POz was the best C1 electrode, 9% had PO4, 7% had PO3, 7% Pz, 2% P1, and 2% Oz. These electrodes were used for all subsequent analyses.

#### Individual alpha frequency computation

Individual alpha frequency computation (IAF) was computed from data from a 500 ms prestimulus window. Each trial was zero-padded (frequency resolution 0.15 Hz), tapered with a Hamming window, and linearly detrended before performing an FFT (Samaha and Postle, 2015). Single-trial power estimates were log10 transformed and IAF was computed as the local maximum in the trial-averaged spectrum within a frequency range from 7 to 14 Hz (see Figure 2).

#### ERP latency measures

We used two different approaches to compute the latency of the early sensory responses (the C1 component and the subsequent N150 component). First we identified the *peak latency* of the C1 and N150. C1 peaks were identified by recording the timing of the local maximum (LVF stimulus) or minimum (UVF stimulus) ERP voltage in a 40-96 ms window after stimulus onset. Due to the large C1 peaks overlapping and influencing the later N150 component, a different window was used for the UVF and LVF N150 peaks. N150 peaks in the LVF were identified from within a 96-230 ms window, and N150 peaks in the UVF from within a 120-230 ms window. Because the peak latency is an arbitrary waveform feature and possibly contaminated with noise, we additionally computed the *onset latency* of each component, measured as the 50% fractional latency (Luck, 2005). Onset latency was computed as the timepoint at which each ERP reached 50% of its peak amplitude value relative to 0 μV. For analyses involving the N150 onset measures, two participants were left out from the attention task and four participants were left out from the task-irrelevant viewing dataset due to positive N150 peak amplitudes. Lastly, we also computed difference scores between the C1 and N150 component latencies to derive a measure of the relative timing between ERP components. Specifically, we subtracted C1 peak latencies from N150 peak latencies and C1 onset latencies from N150 onset latencies separately for UVF and LVF. These difference scores were used to capture the possibility that IAF was related not to the absolute latency of the responses but to the relative latency with which the responses were generated in the visual system. Thus, we derived a total of 12 latency metrics: C1 peak and onset, N150 peak and onset, and N150 - C1 peak and onset difference, each for upper and lower visual field stimuli.

#### Peak frequency of the ERP

Because the detection of ERP components uses some arbitrary waveform features (e.g., peak, or 50% latency) and because the N150 component was difficult to identify clearly for all subjects and visual field locations (see Results), we supplemented our main analysis with the following more agnostic, data driven approach. Based on the fact that the difference between the UVF and LVF stimuli reflect spatially specific responses, we computed the LVF minus UVF difference ERP. This has the added benefit of increasing the signal-to-noise (since the first few deflections in the ERPs have opposing polarities) and also has spectral energy with a peak in the alpha-range. Thus, we also computed the peaks from the FFT of the difference ERP (0 to 500 ms post-stimulus) as a more general measure of spatially-specific neural response latencies, as higher frequency ERPs correspond to smaller delays between peaks. We searched for peaks between 7 and 14 Hz at the best C1 electrode, in keeping with all prior analyses. This approach also has the benefit of summarizing the frequency of an individual’s visual ERP in a single metric (since it collapses across visual fields and we do not need to hand-pick different components and time windows).

#### Statistical analysis

Our primary analysis involved correlating IAF with each measure of component latency using Spearman correlations and data pooled across both studies. We supplemented this analysis with separate Spearman correlations run each task separately. We additionally checked for correlations of latency metrics between upper and lower visual fields as an internal consistency check. Lastly, we compared C1, N150, and the differences in latency metrics (N150 - C1) across the two tasks using independent-samples t-tests to assess any differences in component timing between the two datasets.

## Results

As shown in Figure 2, despite being collected in different labs and with different monitors, stimulus timing, and EEG systems, the ERPs were highly similar. In order to assess any differences in component timings across the two datasets, we ran an independent-samples t-test comparing the various components of interest across the two tasks. Regarding the C1 component, we found no significant task difference for the UVF C1 peak latencies [*t*(40) = −1.05, *p* = 0.30], but we did find a significant task difference for the LVF C1 peak latencies [*t*(40) = −2.12, *p* = 0.04]. An opposite pattern was seen for C1 onset latency such that there *was* a significant task difference for UVF C1 onset latencies [*t*(40) = −3.16, *p* < 0.01] but there was *not* a significant task difference for LVF C1 onset latencies [*t*(40) = −1.88, *p* = 0.07]. This indicates that C1 onset latencies tended to be earlier for the attention task, although this was not replicated across visual fields or latency metrics (e.g., onset versus peak). Regarding N150 latencies, there was a significant task difference in UVF N150 peak latencies [*t*(40) = 2.74, *p* < 0.01], but no significant task difference in UVF N150 onset latencies [*t*(34) = 1.72, *p* = 0.09]. We found no significant differences for either LVF N150 latency measure across tasks [peak: *t*(40) = −0.94, *p* = 0.36; onset: *t*(34) = −0.99, *p* = 0.33]. This indicates that the attention task was associated with earlier onset N150, although only for the UVF and for one latency metric. Regarding the difference in ERP component latencies (N150 - C1) across tasks, results showed a significant task difference for the UVF [peak: *t*(40) = 3.33, *p* < 0.01; onset: *t*(34) = 2.80, *p* < 0.01], but not in the LVF [peak: *t*(40) = −0.32, *p* = 0.75; onset: *t*(34) = −0.60, *p* = 0.55]. Again, these task differences were confined to the UVF. Finally, there was no significant difference in peak alpha frequencies between the two datasets [*t*(40) = 0.08, *p* = 0.94].

We speculate that any differences in ERP component timing across the experiments could be due to the different recording environments and equipment. Specifically, the task-irrelevant viewing paradigm used a CRT monitor which tends to have lower absolute luminance outputs which would affect the absolute contrast of the stimuli. Because our focus is on the C1 component which is generally not very sensitive to task differences, and because any overall difference in component timing should not preclude observing an effect of individual differences, we aggregated data across the two studies for our main analysis. However, we also report correlations for each task separately to assess any task-related differences.

As a sanity check before our main analysis, we examined correlations between the UVF and LVF ERP component timings across subjects, as we would expect the timing of these events to be related. We combined datasets for this analysis, given that we were comparing across subjects. Indeed, the UVF and LVF C1 peak and onset latencies were significantly correlated across visual fields [peak: *r*(40) = 0.39, *p* = 0.01; onset: *r*(40) = 0.36, *p* = 0.02]. There was no significant correlation for the UVF or LVF N150 peak or onset latencies [peak: *r*(40) = 0.21, *p* = 0.18; onset: *r*(34) = 0.29 *p* = 0.09], although the relationships were both positive. We expect more variation in the latency of N150 measures as they are affected differently by the overlap of the polarity-reversing C1 responses for UVF and LVF. As a result, we also see no significant relationship between C1 and N150 peak and onset latency differences [peak: *r*(40) = 0.15, *p* = 0.35; onset: *r*(34) = 0.18, *p* = 0.29]. This result confirms that the C1 shows reasonable within-subject consistency in timing across the two visual field locations.

**Figure 3:**
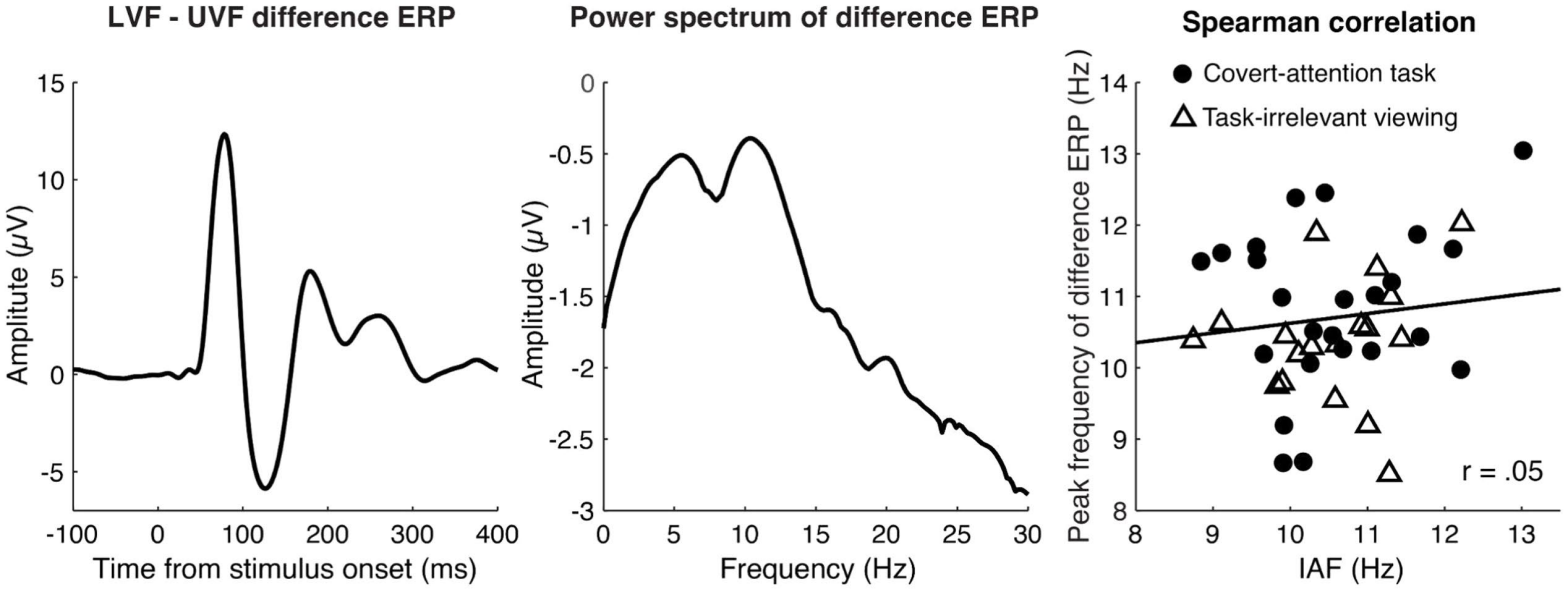
Relationships between IAF and the latencies of ERP components for LVF (blue shapes) and UVF (red shapes) stimuli. Dots represent individual participants from the task-irrelevant viewing dataset and triangles represent participants from the covert attention task. The black lines represent the least-squares fit describing how the onset and peak latencies of C1 and N150 change as IAF changes. **(A)** There were no significant Spearman correlations between IAF and C1 onset latency (left two columns) or N150 onset latency (right two columns). **(B)** There were no significant Spearman correlations between IAF and C1 peak latency (left two columns) or N150 peak latency (right two columns). **(C)** No significant Spearman correlations were found between IAF and the difference in onset latency (left two columns) or peak latency (right two columns).

Our main analysis evaluated the relationship between IAF and C1 latency measures pooling data across tasks (Figure 3). We found no significant correlation between IAF and C1 peak latency for either visual field [UVF: *r*(40) = 0.09, *p* = 0.59; LVF: *r*(40) = 0.08, *p* = 0.61] or C1 onset latency for either visual field [UVF: *r*(40) = −0.08, *p* = 0.62; LVF: *r*(40) = 0.06, *p* = 0.69]. Additionally, there was no significant correlation between N150 peak latency and IAF for either visual field [UVF: *r*(40) = −0.13, *p* = 0.41; LVF: *r*(40) = 0.12, *p* = 0.45], or between N150 onset latency and IAF [UVF: *r*(34) = −0.25, *p* = 0.15; LVF: *r*(34) = 0.10, *p* = 0.51]. Similarly, there was no significant correlation between the differences in peak latencies and IAF for either visual field [UVF: *r*(40) = −0.25, *p* = 0.15; LVF: *r*(40) = 0.08, *p* = 0.65], or the differences in onset latencies and IAF [UVF: *r*(34) = −0.24., *p* = 0.16; LVF: *r*(34) = 0.07, *p* = 0.68].

To determine whether participants within each task showed any different effects, we also report correlations separated by task. For the task-irrelevant viewing paradigm, there were no significant correlations between IAF and any of the ERP components or the latency differences between components in the UVF (Table 1). Additionally, there were no significant correlations between C1 peak latency or C1 onset latency for the LVF (Table 1), but there were significant positive correlations between IAF and N150 peak latency [*r*(22) = 0.52, *p* < 0.01], and N150 onset latency [*r*(18) = 0.47, *p* = 0.04], and subsequently, the difference in peak and onset latencies between the components in the LVF [peak: *r*(22) = 0.43, *p* = 0.03; onset: *r*(18) = 0.50, *p* = 0.02]. For the covert-attention task paradigm, there were also no significant correlations between IAF and any of the ERP components or the latency differences between components in the UVF (Table 1), although a negative correlation between IAF and the C1 peak latency was marginally significant [*r*(16) = −0.45, *p* = 0.06]. For the LVF, no significant correlation was found between IAF and either C1 components, the N150 peak latency, or the difference in latency between component peaks (Table 1). There was a significant negative correlation between IAF and N150 onset latency for the LVF stimuli [*r*(16) = −0.53, *p* = 0.03] as well as between IAF and the difference in latency between component onsets [*r*(14) = −0.53, *p* = 0.04].

**Table 1:** Individual alpha frequency (IAF) was correlated with C1 and N150 ERP peak and onset latencies, as well as the differences between component peak and onset latencies, using a Spearman correlation.

	Task-irrelevant viewing				Covert-attention task			
	Upper visual field		Lower visual field		Upper visual field		Lower visual field	
	*r*	*p*	*r*	*p*	*r*	*p*	*r*	*p*
C1 peak latency	0.15	0.47	0.27	0.19	−0.44	0.06	−0.25	0.31
C1 onset latency	−0.02	0.92	0.08	0.73	−0.38	0.12	−0.10	0.69
N150 peak latency	−0.16	0.46	0.52	0.009*	−0.03	0.90	−0.41	0.09
N150 onset latency	−0.33	0.15	0.47	0.04*	−0.11	0.69	−0.53	0.03*
Peak latency difference	−0.17	0.43	0.43	0.03*	−0.25	0.32	−0.37	0.13
Onset latency difference	−0.30	0.20	0.50	0.02*	0.05	0.87	−0.53	0.04*

For the N150 onset latency and onset latency difference measures, two participants were removed from the covert-attention task and four were removed from the task-irrelevant viewing paradigm. The full dataset was used for all other correlations. Correlations between ERP components and individual alpha frequency (IAF).

* Indicate significant correlations (*p* < 0.05).

Lastly, as a data-driven approach to estimating the relative latency of each participant’s evoked response, we considered the correlation between IAF and the peak frequency of the ERP difference wave (LVF-UVF; see Methods). We found no significant relationship between IAF and peak frequency of the difference ERP waveform for either task [task-irrelevant viewing: *r*(22) = 0.06, *p* = 0.77; covert-attention task: *r*(16) = 0.12, *p* = 0.64], or for the two tasks combined [*r*(40) = 0.05, *p* = 0.73].

## Discussion

Our main analysis found no significant correlations between IAF and any of the ERP peak, onset, or latency difference measures when collapsing across tasks. While we did find a few significant correlations when examining tasks separately, specifically driven by the N150 ERP, these relationships were only seen in one visual field (LVF), and were in different directions for the different tasks, making the results difficult to interpret according to either discrete or rhythmic perception account. We thus interpret these specific and opposing significant correlations as likely reflecting noise (i.e., type 1 error given that many correlations were computed). Our main focus was on the C1 ERP was the component since our stimuli were designed to elicit this response, subjects had clear C1 components, and it is the first visual-evoked response, potentially being most susceptible to modulation by alpha frequency given putative generators in the visual thalamus and primary visual cortex (Hughes and Crunelli, 2005; Lőrincz et al., 2009; Dougherty et al., 2017). However, the lack of a relationship between IAF and C1 peak or onset latency in the main and task-specific analyses suggests that the frequency of alpha is unrelated to the timing with which visual responses first arrive in the primary visual cortex.

We also found that IAF was not reliably predictive of N150 latency, nor the difference between C1 and N150 latency metrics in any of our pooled analysis. However, we did find significant *positive* correlations between IAF and N150 onset latency and IAF and onset latency differences in the LVF for the task-irrelevant viewing paradigm, meaning that as IAF increased across participants, N150 onsets occurred *later* in time and the difference between C1 onset and N150 onset *increased*. However, we found significant *negative* correlations between IAF and these same components in the LVF for the covert-attention task. In other words, as IAF increased across participants, the N150 onset occurred *earlier* in time and the difference between C1 onset and N150 onset *decreased*. It is unclear what theory would predict a different direction of correlation under different task demands, or what theory would predict a correlation between IAF and ERPs in only one visual field, so we do not put much weight on these results, which are also based on smaller sample sizes.

Overall, our findings suggest that alpha frequency does not modulate the timing of neural responses associated with early perceptual processing, an effect we would expect to see if alpha oscillations were indeed responsible for discretizing perceptual events as theorized by the discrete perception account. While it is true that these results do not rule out discrete perception, they suggest that any discretization of percepts that may manifest in behavior likely results from later perceptual processing, as opposed to through these early sensory ERP components. Additionally, a previous analysis of this same dataset showed an effect of pre-stimulus alpha phase on the amplitude, but not timing, of early sensory activity (Dou et al., 2022). Thus, there is little evidence to suggest that alpha oscillations are responsible for driving changes in the latency of neural events, as suggested by the discrete perception account. However, the prediction that alpha oscillations modulate the strength of sensory responses is supported by prior work (Dou et al., 2022).

**Figure 4:**
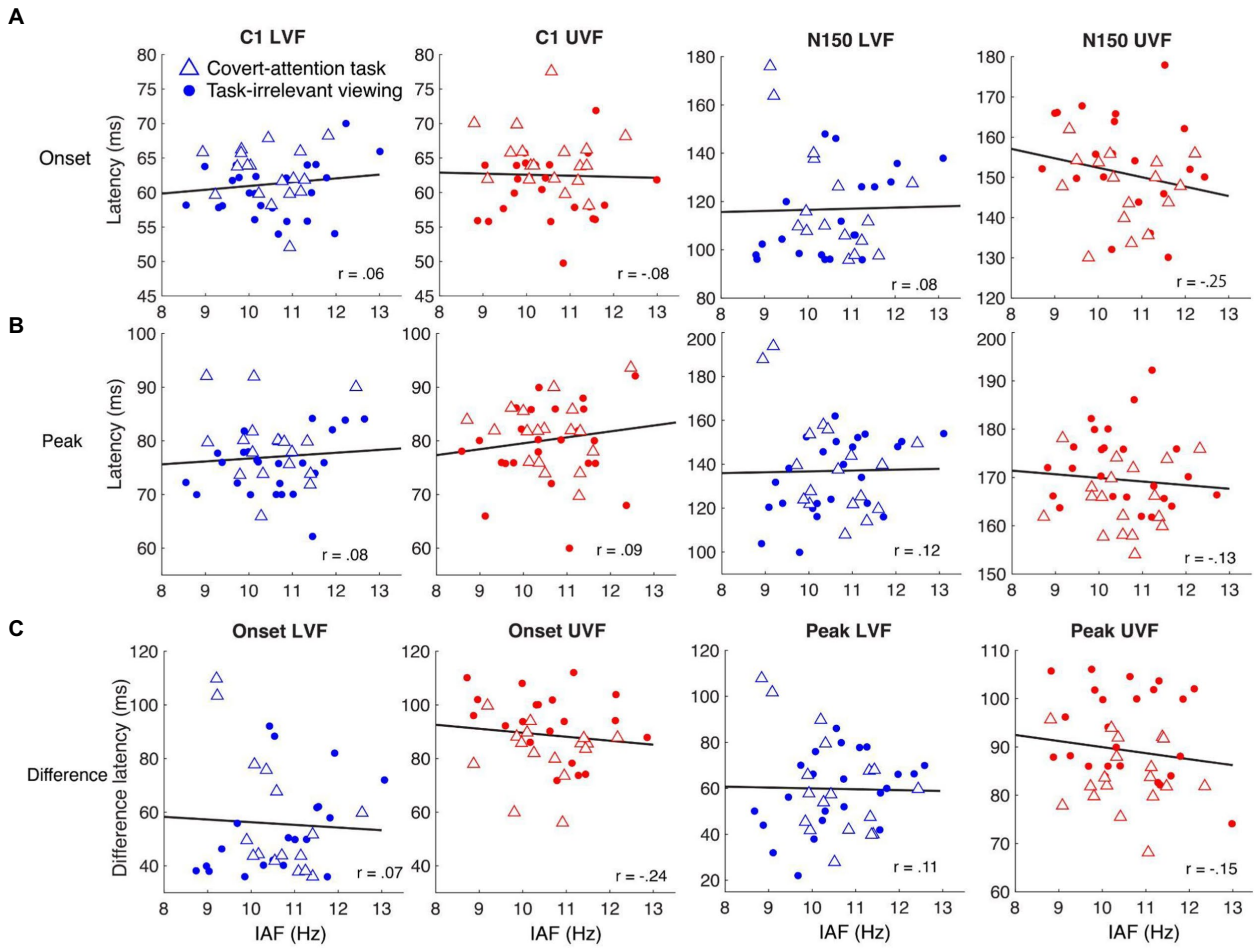
Peak Frequency of the LVF minus UVF difference ERP for the combined tasks. The left panel shows the grand average difference ERP (LVF-UVF), which captures spatially-specific activity and boosts the signal-to-noise ratio in our data. The middle panel shows the grand averaged power spectrum of the difference ERP within the post-stimulus time window of 0 to 500 ms, revealing a clear alpha peak. The right panel illustrates the relationship between peak frequency of the difference ERP (computed between 7 and 14 Hz) and IAF. Dots represent individual participants from the task-irrelevant viewing dataset and triangles represent participants from the covert attention task. The black lines represent the least-squares fit. A Spearman correlation showed no significant relationship between these two peak frequencies for the aggregated data or when each task was analyzed separately.

The lack of a relationship between IAF and the peak frequency of the ERP difference waveform (Figure 4) also supports the lack of a relationship between alpha frequency and neural response latencies. One reason for the null effect could be related to the stimulus used here. A seminal study by VanRullen and Macdonald (2012) showed that, when participants viewed a stimulus that randomly modulated in luminance at 160 Hz over a 6 s period, there was a cross-correlation between the EEG signal over occipital electrodes and the luminance values that lasted up to 1 s and which, importantly, fluctuated at an alpha frequency (so-called alpha “echoes”). This implies that a unit change in luminance causes a long-lasting reverberation in the alpha frequency. Interestingly, the frequency of an individual’s alpha echo was found to strongly correlate with their resting IAF (VanRullen and Macdonald, 2012), which would imply that the timing of neural responses to a luminance change is related to IAF.

We suggest two possible interpretations of our null results in light of the VanRullen & Macdonald finding. First, our stimuli were defined by their contrast rather than their luminance (each increase in brightness was canceled out by a decrease in brightness elsewhere in the stimulus). Thus, it remains possible that the timing of luminance responses is perhaps related to alpha frequency in a way that contrast responses is not. Second, we measured ERP onset metrics, not alpha echoes. ERPs likely reflect a mixture of some steady-state response (as in the alpha echo) and various onset and offset responses caused by the sudden appearance and disappearance of the stimulus. Thus it remains possible that only the steady-state component of the visual response is related to alpha frequency, but not the onset or offset transients. This would imply that the alpha echo approach measures a qualitatively different aspect of visual processing than ERPs.

Our results also have implications for the idea that visual ERP components are generated by a phase-rest of ongoing oscillations (Gruber et al., 2005; Klimesch et al., 2007). Although our conclusions are restricted to the alpha-band, the lack of correlation speaks against the idea that stimulus onset resets ongoing alpha oscillations and that this is what produces (or contributes) to ERP generation. If the C1 or N150 were generated by a phase reset, we would expect a strong correlation between the frequency of the oscillations being reset and the timing of the ERP components, which was not found. Instead, it is likely that these early visual components reflect additive neural activity that sums with ongoing or background neural oscillations (Iemi et al., 2019).

Future research could further assess whether IAF is related to the latency of other early sensory responses, such as the P1 and N1, as these ERPs were not clear in our datasets due to the high-amplitude C1 response. It is possible that there may be instances where discrete perception occurs and different ways that discrete perception may manifest. However, given that alpha frequency was not related to the latency of the earliest visual ERP (C1), our findings are inconsistent with the notion that alpha is modulating the timing of afferent visual responses.

## Data availability statement

The datasets presented in this study can be found in online repositories/Supplementary material. The names of the repository/repositories and accession number(s) can be found in the article.

## Ethics statement

The studies involving human participants were reviewed and approved by University of California Santa Cruz Institutional Review Board. The patients/participants provided their written informed consent to participate in this study.

## Author contributions

JS developed the idea for this project, and was instrumental in guiding data analysis and providing feedback on the manuscript. WD and AM carried out the data analysis. AM was responsible for data collection in the visual attention task and primarily wrote the manuscript, while WD primarily developed figures for the manuscript. All authors contributed to the article and approved the submitted version.

## Conflict of interest

The authors declare that the research was conducted in the absence of any commercial or financial relationships that could be construed as a potential conflict of interest.

## Publisher’s note

All claims expressed in this article are solely those of the authors and do not necessarily represent those of their affiliated organizations, or those of the publisher, the editors and the reviewers. Any product that may be evaluated in this article, or claim that may be made by its manufacturer, is not guaranteed or endorsed by the publisher.
