# Supplementary material for: Individual alpha frequency appears unrelated to the latency of early visual responses
Source: Front Neurosci. 2023 Apr 11;17:1118910. doi: 10.3389/fnins.2023.1118910 (PMC10126513; doi:10.3389/fnins.2023.1118910)
Supplement: Supplementary file 1 [file Data_Sheet_1.docx]

APPENDIX

​​

| **Subject #** | **Task** | **IAF** | **LVF C1 peak** | **UVF C1 peak** | **LVF C1 onset** | **UVF C1 onset** | **LVF N150 peak** | **UVF N150 peak** | **LVF N150 onset** | **UVF N150 onset** | **LVF peak diff.** | **UVF peak diff.** | **LVF onset diff.** | **UVF onset diff.** |
| --- | --- | --- | --- | --- | --- | --- | --- | --- | --- | --- | --- | --- | --- | --- |
| **1** | **TI** | **11.36** | 76 | 86 | 64 | 72 | 154 | 168 | 126 | 146 | 78 | 82 | 62 | 74 |
| **2** | **TI** | **10.57** | 70 | 72 | 54 | 56 | 150 | 176 | 146 | 156 | 80 | 104 | 92 | 100 |
| **3** | **TI** | **8.97** | 72 | 80 | 58 | 64 | 104 | 176 | 98 | 166 | 32 | 96 | 40 | 102 |
| **4** | **TI** | **11.16** | 76 | 80 | 56 | 58 | 134 | 162 | 106 | 136 | 58 | 82 | 50 | 78 |
| **5** | **TI** | **9.97** | 72 | 76 | 62 | 62 | 138 | 176 | 98 | 156 | 66 | 100 | 36 | 94 |
| **6** | **TI** | **11.96** | 82 | 76 | 54 | 58 | 148 | 176 | 136 | 152 | 66 | 100 | 82 | 94 |
| **7** | **TI** | **8.77** | 70 | 78 | 58 | 56 | 120 | 166 | 96 | 152 | 50 | 88 | 38 | 96 |
| **8** | **TI** | **9.77** | 82 | 76 | 64 | 60 | 152 | 182 | 120 | 168 | 70 | 106 | 56 | 108 |
| **9** | **TI** | **11.56** | 74 | 76 | 60 | 56 | 116 | 164 | 96 | 130 | 42 | 88 | 36 | 74 |
| **10** | **TI** | **10.37** | 70 | 80 | 58 | 62 | 146 | 166 | NaN | NaN | 76 | 86 | NaN | NaN |
| **11** | **TI** | **10.77** | 76 | 86 | 62 | 64 | 140 | 186 | 112 | 154 | 64 | 100 | 50 | 90 |
| **12** | **TI** | **10.96** | 70 | 60 | 56 | 50 | 148 | 162 | 106 | 144 | 78 | 102 | 50 | 94 |
| **13** | **TI** | **9.77** | 78 | 86 | 62 | 66 | 100 | 172 | NaN | NaN | 22 | 86 | NaN | NaN |
| **14** | **TI** | **9.37** | 76 | 76 | 58 | 58 | 132 | 164 | 104 | 150 | 56 | 88 | 46 | 92 |
| **15** | **TI** | **10.57** | 72 | 80 | 58 | 60 | 124 | 166 | 98 | 132 | 52 | 86 | 40 | 72 |
| **16** | **TI** | **12.16** | 84 | 68 | 70 | 58 | 150 | 170 | 128 | 162 | 66 | 102 | 58 | 104 |
| **17** | **TI** | **10.17** | 70 | 82 | 60 | 62 | 120 | 176 | NaN | NaN | 50 | 94 | NaN | NaN |
| **18** | **TI** | **12.96** | 84 | 92 | 66 | 62 | 154 | 166 | 138 | 150 | 70 | 74 | 72 | 88 |
| **19** | **TI** | **8.97** | 78 | 66 | 64 | 56 | 122 | 172 | 102 | 166 | 44 | 106 | 38 | 110 |
| **20** | **TI** | **11.56** | 62 | 82 | 62 | 56 | 122 | 166 | 96 | 166 | 60 | 84 | 42 | 102 |
| **21** | **TI** | **10.17** | 76 | 86 | 56 | 64 | 122 | 170 | 96 | 150 | 46 | 84 | 40 | 86 |
| **22** | **TI** | **11.36** | 84 | 88 | 64 | 66 | 152 | 192 | 126 | 178 | 68 | 104 | 62 | 112 |
| **23** | **TI** | **9.97** | 78 | 78 | 62 | 64 | 116 | 180 | NaN | NaN | 38 | 102 | NaN | NaN |
| **24** | **TI** | **10.37** | 76 | 90 | 60 | 64 | 162 | 180 | 148 | 164 | 86 | 90 | 88 | 100 |
| **25** | **CA** | **9.97** | 74 | 76 | 64 | 66 | 128 | 158 | 116 | 154 | 54 | 82 | 50 | 88 |
| **26** | **CA** | **9.77** | 82 | 86 | 66 | 66 | 140 | 168 | NaN | NaN | 58 | 82 | NaN | NaN |
| **27** | **CA** | **10.17** | 74 | 76 | 60 | 64 | 154 | 170 | 138 | 150 | 80 | 94 | 78 | 86 |
| **28** | **CA** | **10.57** | 80 | 82 | 68 | 60 | 138 | 158 | 110 | 140 | 58 | 76 | 42 | 80 |
| **29** | **CA** | **9.17** | 92 | 82 | 60 | 62 | 194 | 178 | 164 | 162 | 102 | 96 | 104 | 100 |
| **30** | **CA** | **11.36** | 74 | 70 | 62 | 58 | 114 | 162 | 98 | 144 | 40 | 92 | 36 | 86 |
| **31** | **CA** | **9.97** | 80 | 86 | 66 | 70 | 122 | 166 | 110 | 130 | 42 | 80 | 44 | 60 |
| **32** | **CA** | **10.17** | 92 | 82 | 64 | 62 | 158 | 174 | 140 | 156 | 66 | 92 | 76 | 94 |
| **33** | **CA** | **10.77** | 80 | 90 | 52 | 78 | 108 | 158 | 96 | 134 | 28 | 68 | 44 | 56 |
| **34** | **CA** | **11.16** | 78 | 82 | 62 | 64 | 126 | 166 | 106 | 150 | 48 | 84 | 44 | 86 |
| **35** | **CA** | **10.96** | 80 | 74 | 60 | 62 | 122 | 154 | 98 | 136 | 42 | 80 | 38 | 74 |
| **36** | **CA** | **10.96** | 76 | 86 | 62 | 66 | 144 | 172 | NaN | NaN | 68 | 86 | NaN | NaN |
| **37** | **CA** | **10.57** | 66 | 74 | 58 | 62 | 156 | 162 | 126 | 144 | 90 | 88 | 68 | 82 |
| **38** | **CA** | **12.36** | 90 | 94 | 68 | 68 | 150 | 176 | 128 | 156 | 60 | 82 | 60 | 88 |
| **39** | **CA** | **11.36** | 80 | 82 | 66 | 66 | 120 | 174 | 104 | 154 | 40 | 92 | 38 | 88 |
| **40** | **CA** | **9.97** | 78 | 82 | 64 | 64 | 124 | 166 | 108 | 154 | 46 | 84 | 44 | 90 |
| **41** | **CA** | **11.36** | 72 | 78 | 60 | 64 | 140 | 160 | 112 | 148 | 68 | 82 | 52 | 84 |
| **42** | **CA** | **8.97** | 80 | 84 | 66 | 70 | 188 | 162 | 176 | 148 | 108 | 78 | 110 | 78 |

*Appendix A*. The latencies in milliseconds of each ERP component measure, separated by tasks, where ‘TI’ indicates task-irrelevant data and ‘CA’ indicates covert-attention data. Participants who had positive-going N150 peaks were left out of N150 onset latency analyses and the onset latency difference analyses and show NaN for these measures.
